# Histone deacetylation of *NIS* promoter underlies BRAF V600E-promoted *NIS* silencing in thyroid cancer

**DOI:** 10.1530/ERC-13-0399

**Published:** 2014-04

**Authors:** Zongjing Zhang, Dingxie Liu, Avaniyapuram Kannan Murugan, Zhimin Liu, Mingzhao Xing

**Affiliations:** 1 Division of Endocrinology, Diabetes, and Metabolism, Laboratory for Cellular and Molecular Thyroid ResearchJohns Hopkins University School of Medicine1830 East Monument Street, Suite 333, Baltimore, Maryland, 21287USA; 2 Department of Endocrinology and MetabolismChangzheng Hospital, the Second Military Medical UniversityShanghaiChina

**Keywords:** thyroid cancer, *NIS* gene, *BRAF* V600E mutation, histone acetylation, histone deacetylation, radioiodine

## Abstract

The *BRAF* V600E mutation causes impaired expression of sodium iodide symporter (NIS) and radioiodine refractoriness of thyroid cancer, but the underlying mechanism remains undefined. In this study, we hypothesized that histone deacetylation at the *NIS* (*SLC5A5*) promoter was the mechanism. Using the chromatin immunoprecipitation approach, we examined histone acetylation status on the lysine residues H3K9/14, H3K18, total H4, and H4K16 at the *NIS* promoter under the influence of BRAF V600E. We found that expression of stably or transiently transfected BRAF V600E inhibited *NIS* expression while the deacetylase inhibitor SAHA stimulated *NIS* expression in PCCL3 rat thyroid cells. Although BRAF V600E enhanced global histone acetylation, it caused histone deacetylation at the *NIS* promoter while SAHA caused acetylation in the cells. In human thyroid cancer BCPAP cells harboring homozygous *BRAF* V600E mutation, BRAF V600E inhibitor, PLX4032, and MEK inhibitor, AZD6244, increased histone acetylation of the *NIS* promoter, suggesting that BRAF V600E normally maintained histone in a deacetylated state at the *NIS* promoter. The regions most commonly affected with deacetylation by BRAF V600E were the transcriptionally active areas upstream of the translation start that contained important transcription factor binding sites, including nucleotides −297/−107 in the rat *NIS* promoter and −692/−370 in the human *NIS* promoter. Our findings not only reveal an epigenetic mechanism for BRAF V600E-promoted *NIS* silencing involving histone deacetylation at critical regulatory regions of the *NIS* promoter but also provide further support for our previously proposed combination therapy targeting major signaling pathways and histone deacetylase to restore thyroid gene expression for radioiodine treatment of thyroid cancer.

## Introduction

*BRAF* V600E mutation is the most common genetic alteration in thyroid cancer, particularly papillary thyroid cancer (PTC; Xing 2005), and plays an important role in thyroid tumorigenesis through aberrantly activating the RAS-BRAF-MEK-MAP kinase (MAPK) pathway (Xing 2013). Numerous studies around the world have demonstrated an aggressive role of this mutation in the progression and aggressiveness, including increased recurrence, of PTC (Xing 2007*a*, Kim *et al*. 2012, Xing *et al*. 2013*a*). A strong association of BRAF V600E mutation with PTC-related mortality has also been recently demonstrated in a large multicenter study (Xing *et al*. 2013*b*). Unique molecular derangements caused by BRAF V600E/MAPK pathway are the mechanisms for this aggressive role of BRAF V600E (Xing 2013). Among these is the impairment of the iodide-handling machinery of thyroid cells, as reflected by the initial observation of a strong association of BRAF V600E mutation with the loss of radioiodine avidity in PTC (Xing *et al*. 2005), a phenomenon that has been confirmed in numerous subsequent studies (Xing 2007*a*, Kim *et al*. 2012, Xing *et al*. 2013*a*). This phenomenon is clinically relevant, as it suggests an increased risk of radioiodine treatment failure of BRAF V600E-positive PTC, providing an explanation for the association of this mutation with increased disease recurrence and patient mortality of PTC. Correspondingly, numerous studies have reported an association of BRAF V600E with decreased or lost expression of thyroid iodide-handling genes in PTC, particularly sodium iodide symporter (NIS; Xing 2007*a*, Kim *et al*. 2012, Xing *et al*. 2013*a*), which is normally localized in the basal membrane of thyroid cells and function to transport iodide from the blood stream to the intracellular compartment of the thyroid cell for thyroid hormone synthesis. Our previous studies have shown that introduced expression of BRAF V600E in thyroid cells could induce the silencing of various thyroid iodide-handling genes, most prominently the *NIS* gene (Liu *et al*. 2007). BRAF V600E expression could also cause mislocalization of NIS in the cytoplasm in addition to its decreased expression in thyroid cells (Riesco-Eizaguirre *et al*. 2006). In *in vitro* cell line assays, inhibition of the BRAF V600E/MEK pathway or silencing of BRAF V600E expression could restore the expression of thyroid genes, particularly *NIS* in thyroid cells (Liu *et al*. 2007), which provided important therapeutic implications for targeting the BRAF V600E/MAPK pathway to restore thyroid gene expression and radioiodine avidity of radioiodine-refractory thyroid cancer. This *in vitro* finding of the reversibility of BRAF V600E-induced silencing of thyroid genes was recently reproduced in an *in vivo* transgenic mouse model (Chakravarty *et al*. 2011). However, even given all these exciting achievements, a critical question remains open as to what the molecular mechanism underlies the downregulation of thyroid genes by the BRAF V600E/MAPK pathway.

Histone acetylation is an important epigenetic event that plays a fundamental role in the regulation of gene expression (Li *et al*. 2007, Dhall & Chatterjee 2011, Woo & Li 2012, Horikoshi 2013). It typically occurs at multiple lysine residues in the N-terminal domain of H3 (Lys 4, 9, 14, 18, 23, and 27) and H4 (Lys 5, 8, 12, and 16) (Kuo *et al*. 1996, Dhall & Chatterjee 2011). Histone acetylation and deacetylation are associated with gene transcriptional activation and repression, respectively, representing a fundamental mechanism in aberrant gene activities that promote human tumorigenesis (Barneda-Zahonero & Parra 2012, Horikoshi 2013). This has been particularly well established for the acetylation sites H3K9/14, H3K18, and H4K16 of histones.

Histone acetylation and deacetylation are reversible and are catalyzed by histone acetyltransferases and histone deacetylases (HDACs) respectively. It has been recently hypothesized that BRAF V600E/MAPK pathway might downregulate histone acetylation as a mechanism involving aberrant silencing of thyroid iodide-handling genes in thyroid cancer (Xing 2013). In fact, we demonstrated that the inhibition of HDAC could synergize the inhibitors of BRAF V600E/MAPK pathways to robustly increase thyroid gene expression and radioiodine uptake in thyroid cancer cells (Hou *et al*. 2010) and even in certain nonthyroid epithelial cancer cells (Hou *et al*. 2009, Liu & Xing 2012). This result not only had strong implications for the therapeutic use of HDAC inhibitors to restore radioiodine avidity in thyroid cancer, but was also consistent with the hypothesis of impairment of histone acetylation as a mechanism in BRAF V600E/MAPK pathway-induced thyroid gene silencing. Puzzlingly, however, one study demonstrated that BRAF V600E was in fact associated with increased global histone acetylation in thyroid tumor tissues and in thyroid cell lines expressing BRAF V600E (Puppin *et al*. 2011). This finding was apparently inconsistent with the above hypothesis. To solve this puzzle, in this study, we tested the possibility that BRAF V600E/MAPK pathway might specifically downregulate histone acetylation at critical regulatory regions of the gene promoter. To test this possibility, we have particularly focused on the *NIS* gene in this study as it is the most important gene for thyroid uptake of iodide.

## Materials and methods

### Cell culture and reagents

Rat thyroid PCCL3 cell line and its transfectants were cultured and maintained at 37 °C in H4 complete medium of Coon's modification of Ham's F-12 (F6636, Sigma) supplemented with 5% CO_2_, 5% fetal bovine serum, 1 mIU/ml TSH, 5 μg/ml apo-transferrin, 10 μg/ml insulin, and 10 nM hydrocortisone. PCCL3/BRAF cells conditionally expressed BRAF^V600E^ or WT-BRAF induced by 1 μg/ml doxycycline (DOX; Sigma) for 48 h. The HDAC inhibitor SAHA (Sigma) at 0.5 μM was used to treat cells in some experiments. DMSO or PBS was used as a vehicle control.

Noninducible parental rat PCCL3 cells were transiently transected with various BRAF protein-expressing plasmid constructs using the Lipofectamine 2000 transfection reagent following the manufacturer's instructions (Invitrogen, Life Technologies) and the cells were harvested 48 h after transfection.

The human thyroid cancer cell line, BCPAP, was cultured in RPMI 1640 supplemented with 2 mM l-glutamine, 10% fetal bovine serum, MEM nonessential amino acids (#25-005-Cl, 100×), and 1 mM sodium pyruvate (#25-000-Cl) (all from Coring, Cellgro, Manassas, VA, USA). AZD6244 (Selleck Chemicals, Houston, TX, USA) and PLX4032 (Plexxikon, Inc., Berkeley, CA, USA) were dissolved in DMSO and used to treat cells at 1 μM in some experiments.

### Western blotting

The cells were lysed in RIPA lysis buffer (RIPA) (sc-24948, Santa Cruz Biotechnology) containing standard protease inhibitor cocktails. After measurement and adjustment of protein concentration using the Bio-Rad Dc Protein Assay, 15 μg of each sample was separated on 10% SDS–PAGE, transferred onto PVDF membrane (Amersham Pharmacia Biotech), and blocked with 5% blotting milk (170-6404, Blotting Grade Blocker, Bio-Rad) for 1 h. The following primary antibodies were used for immunoblotting: anti-c-Myc (9E10) (sc-40, Santa Cruz Biotechnology), anti-Raf-B (C-19) (sc-166, Santa Cruz Biotechnology), anti-p-ERK (E-4) (sc-7383, Santa Cruz Biotechnology), anti-β actin (I-19) (sc-1616, Santa Cruz Biotechnology), and anti-H3K9/14 (06-599B, Millipore, Billerica, MA, USA). The cells were incubated overnight with primary antibodies at 4 °C. After washing with TBST three times, each for 5 min, the blots were incubated with HRP-linked anti-mouse or anti-rabbit second antibody for 2 h at room temperature, followed by three 5-min washings. Protein bands in the membrane were visualized with ECL reaction reagents (Amersham Pharmacia Biotech).

### Total RNA isolation, RT-PCR, and quantitative PCR

Total cellular RNA was isolated in 500 μl of Trizol reagent (15596-018, Invitrogen) following the manufactures' instructions. Two microgram of total RNA was reverse-transcribed to DNA using the Script cDNA Synthesis Kit (170-8891, Bio-Rad Laboratories). Real-time PCR was performed using SYBR Green Supermix (Bio-Rad Laboratories) with 50× ROX (54881, Invitrogen) on the ABI 7900HT PCR system (Applied Biosystems). β-actin was run in parallel for quality control. The RT-PCR primers and procedures for rat *NIS* and β-actin genes were as described previously (Liu *et al*. 2007).

### Chromatin immunoprecipitation assay

Chromatin immunoprecipitation (ChIP) assay was performed using the Magna ChIP A kit (17-408, Millipore) according to the manufacturers' protocol as described previously (Liu & Xing 2012). Briefly, protein–DNA was cross-linked by incubating cells with diluted formaldehyde for 10 min. Glycine was then added for 5 min to quench unreacted formaldehyde. After washing with PBS twice, cells were lysed and sonicated ten times each for 10 s with 20 s rest between pulses at a 25% pulse power using the Branson Sonifier (150D Liquid Processor). Chromatin was sheared to lengths between 200 and 1000 bp, mostly between 200 and 500 bp. Cross-linked protein–DNA complexes were incubated overnight at 4 °C with anti-histone acetylation antibodies, including anti-acetylated H3K9/14 (06-599B, Millipore), anti-acetylated H3K18 (9675s, Cell Signaling), anti-acetylated H4 (17-630, Millipore) (the acetyl-histone H4 antiserum was made against a peptide corresponding to amino acids 2–19 of tetrahymena histone H4, which is conserved in eukaryotes and contains the four histone acetylation sites H4K5, H4K8, H4K12, H4K16), anti-acetylated H4K16 (17-10101, Millipore), normal rabbit IgG (PP64B), and fully suspended protein A magnetic beads. After washing protein A beads–antibody/chromatin complexes with 800 μl washing buffer, cross-linked protein–DNA complexes were reversed by incubating with proteinase K at 62 °C for 3 h with gentle shaking. DNA was purified using spin columns. Seven pairs of rat *NIS* primers and five pairs of human *NIS* primers, corresponding to the defined regions of the NIS promoters (Table 1), were used to perform real-time quantitative PCR (qPCR) to detect DNA fragments obtained from ChIP. Real-time qPCR was carried out with an initial denaturation at 95 °C for 10 min, followed by 50 cycles of denaturation at 95 °C for 30 s, and annealing and extension at 60 °C for 1 min. Data were normalized using the β-actin gene. The results of ChIP assays were expressed as the fraction of the input DNA. Threshold cycles (*C*t) were determined for ChIP samples and the input DNA, and the relative amount of immunoprecipitated DNA (% ChIP signal per input DNA) was calculated as 100^2Δ*C*t^ (Puppin *et al*. 2012).

### Gene promoter area prediction

To identify critical regulatory regions of the *NIS* promoter for histone acetylation studies, we carried out prediction analyses for the promoter transcriptional activities and response elements of rat and human *NIS* genes and designed the primers for these regions as presented in Table 1 in the ChIP studies. The following online tools were used for this purpose:

1. For promoter activity: http://tools.igsp.duke.edu/generegulation/McPromoter/;
2. For transcription factor-binding site: http://www.cbil.upenn.edu/cgi-bin/tess/tess;
3. For CpG island: http://www.uscnorris.com/cpgislands2/cpg.aspx;
4. For TATA box: http://zeus2.itb.cnr.it/∼webgene/wwwHC_tata.html.

### Statistical analysis

The data presented are representatives of at least three similar experiments. The differences between groups of at least three experiments were analyzed by *t*-test. A *P* value <0.05 was considered statistically significant.

## Results

### BRAF V600E suppressed *NIS* gene expression in rat thyroid cells

**Figure 1:**
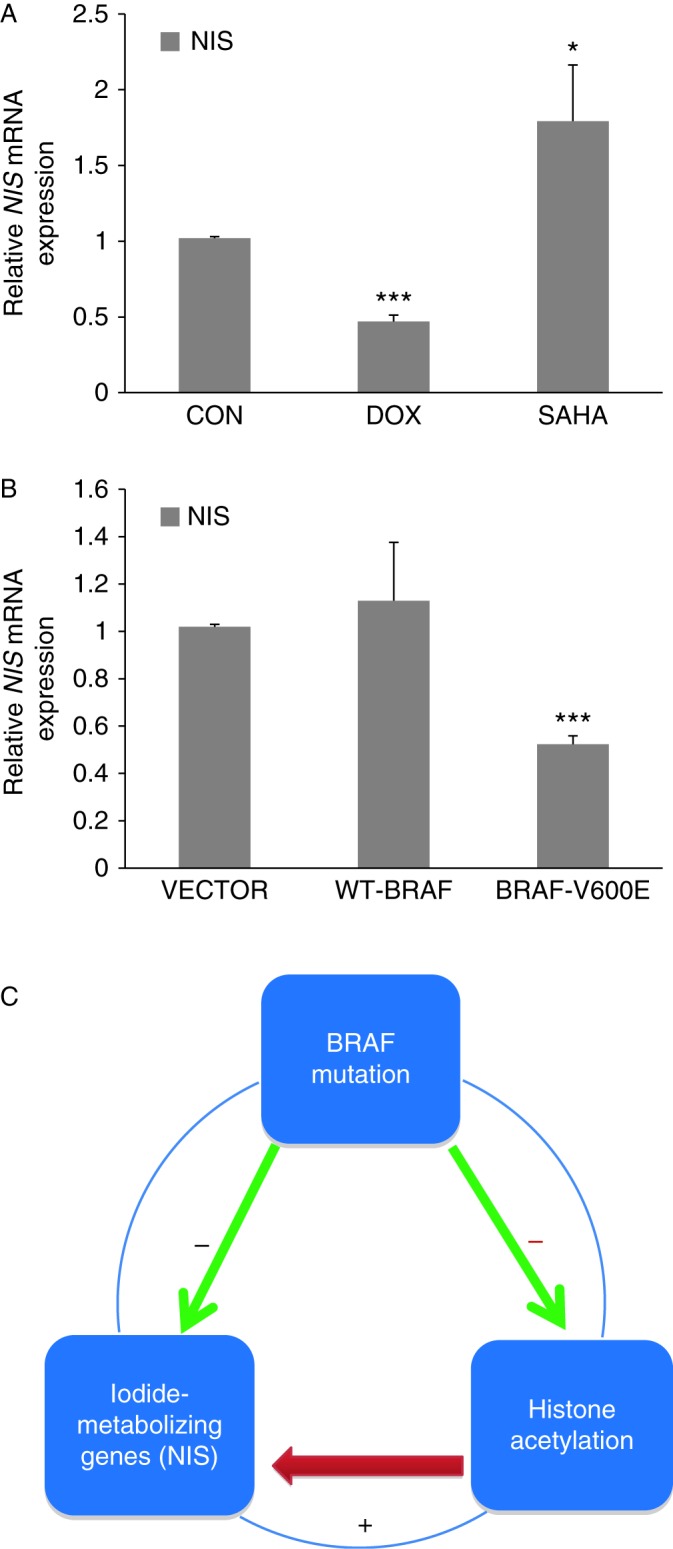
Effect of BRAF V600E on the expression of the *NIS* gene in rat thyroid cells. (A) In PCCL3/BRAF cells, DOX at 1 μg/ml induced BRAFV600E expression and *NIS* gene mRNA expression was correspondingly decreased by 54% in 48 h. The histone deacetylases inhibitor, SAHA, at 0.5 μM increased *NIS* mRNA expression in PCCL3/BRAF cells. (B) PCCL3 cells transiently transfected with BRAF V600E showed a decrease in NIS expression by 48% in 48 h compared with the empty vector, and WT-BRAF did not have effect. (C) Hypothesized mechanism for *BRAF* V600E mutation-induced downregulation of *NIS* gene expression, in which *BRAF*V600E downregulated histone acetylation at the *NIS* promoter and decreased *NIS* expression. Each bar represents the mean value ±s.e.m. of three different experiments. CON, control; DOX, doxycycline. *P<0.05, ***P<0.001.

We previously demonstrated that induced expression of BRAF V600E in PCCL3/BRAF thyroid cells for 6 days or 30 days resulted in dramatic inhibition of the expression of thyroid genes, particularly *NIS* (Liu *et al*. 2007). In this study, we reproduced this result by showing that BRAF V600E could decrease NIS expression even after a 48-h DOX-induced expression of BRAF V600E in PCCL3 cells stably transfected with this oncoprotein (Fig. 1A). In contrast, treatment of cells with the HDAC inhibitor, SAHA, could increase the expression of *NIS*. To further confirm the specific effect of BRAF B600E on *NIS* expression, we performed transient expression of BRAF proteins in PCCL3 cells. In comparison with the empty vector transfection, transient expression of BRAF V600E for 48 h significantly decreased NIS expression in PCCL3 cells, whereas expression of the WT-BRAF had no effect on NIS expression (Fig. 1B). The effects of BRAF V600E expression and HDAC inhibition on *NIS* expression were clearly in opposite directions. Give these results and our previously demonstrated reversibility of BRAF V600E-induced *NIS* suppression (Liu *et al*. 2007) as well as the fact that histone acetylation and deacetylation are rapid reversible biochemical modifications, we believed, as recently hypothesized (Xing 2013), that BRAFV600E/MAPK pathway suppressed the expression of *NIS* by downregulating histone acetylation (Fig. 1C).

### BRAF V600E increased global histone acetylation in thyroid cells

**Figure 2:**
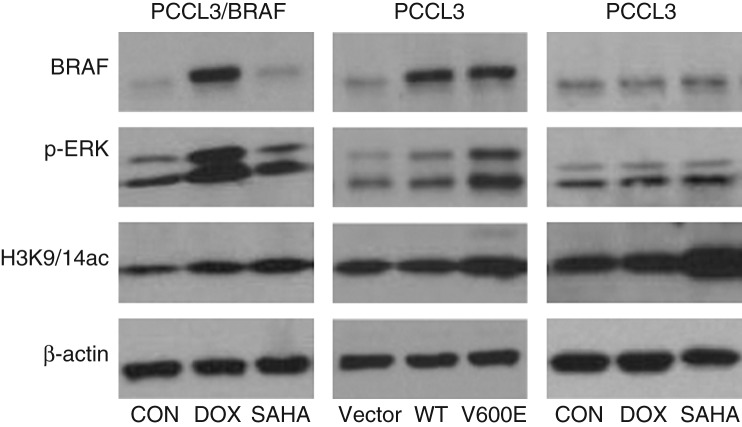
Global histone acetylation changes in PCCL3/BRAF and PCCL3 cells upon various treatments. 10^6^ cells were planted in 12-well plates in each condition. PCCL3/BRAF cells were treated with 1 μg/ml DOX to induce BRAF V600E expression (left), PCCL3 cells were transiently expressed with BRAF V600E (middle), and PCCL3 cells were treated with DOX at 1 μg/ml or SAHA at 0.5 μM (right), followed by cell lysis and protein preparation for western-blotting 48 h later. BRAF V600E expression was successfully induced, accompanied by P-ERK activation and increase in global H3K9/14 acetylation. SAHA at 0.5 μM increased global histone H3K9/14 acetylation in both PCCL3/BRAF cells and WT PCCL3 cells. PCCL3 cells treated with DOX at 1 μg/ml did not show change in histone H3K9/14 acetylation, unlike treatment with SAHA. CON, control; DOX, doxycycline; p-ERK, phosphorylated ERK; H3K9/14ac, acetylated H3K9/14.

As an initial step to test our hypothesis, we first examined the effect of BRAF V600E on the overall global histone acetylation in thyroid cells. At 48 h of induction by DOX, abundant expression of stably transfected BRAF V600E was induced, accompanied by a sharp increase in the phosphorylation of ERK (p-ERK; Fig. 2, left panel). Contrary to our expectation based on our hypothesis, the global histone H3K9/14 acetylation of cells was increased following BRAF V600E expression. This was similar to the increased global histone H3K9/14 acetylation induced by treating cells with the HDAC inhibitor, SAHA (Fig. 2, right and left panels). SAHA did not have effect on the expression of BRAF or p-ERK. These results reproduced a similar previous finding of the upregulation of global histone acetylation by stable expression of BRAF V600E in rat thyroid cells (Puppin *et al*. 2011). Transient expression of BRAF V600E for 48 h also increased p-ERK accompanied by increased global histone acetylation, while expression of WT-BRAF did not have such effects (Fig. 2, middle panel), confirming the specific effect of BRAF V600E on histone acetylation. Unlike SAHA, DOX itself had no effect on histone acetylation in PCCL3 cells (Fig. 2, right panel), demonstrating that the effect of DOX-induced expression of stably transfected BRAF V600E on histone acetylation represented a real effect of BRAF V600E, but not that of DOX. These results on the effects of BRAF V600E on global histone acetylation did not seem to agree with our hypothesis that BRAF V600E downregulates thyroid genes by causing histone deacetylation. We then hypothesized that perhaps, in contrary to the global histone acetylation, histone acetylation at critical regions of the promoter of *NIS* gene might be specifically downregulated by BRAF V600E/MAPK pathway, hence negatively affecting the expression of *NIS*. We hereafter focused on testing this possibility.

### Suppression of H3K9/14 acetylation of histone at the *NIS* promoter by BRAF V600E

**Figure 3:**
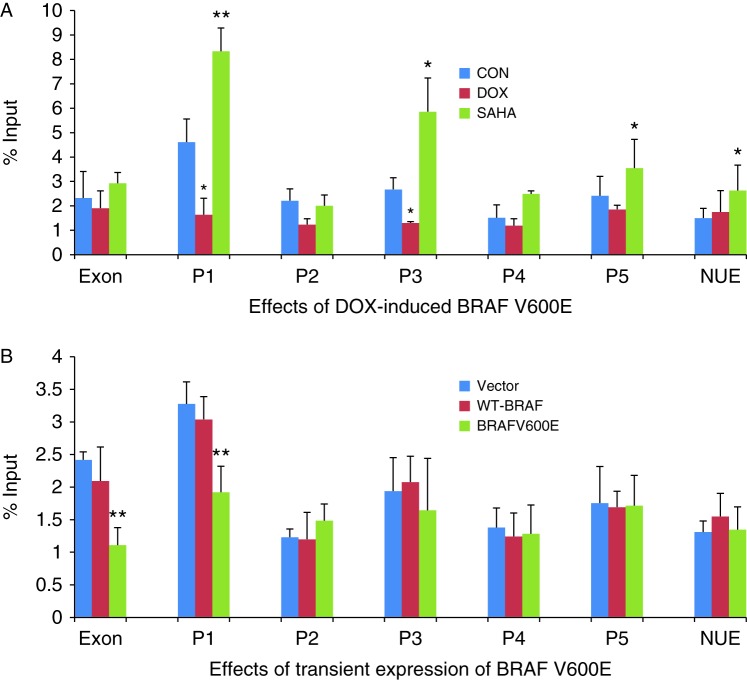
Effect of BRAF V600E on H3K9/14 acetylation in the rat *NIS* promoter in rat thyroid cells. (A) In PCCL3/BRAF cells, DOX at 1 μg/ml induced BRAF V600E expression after 48 h, accompanied by decrease in H3K9/14 acetylation in the regions P1, P2, and P3 of the *NIS* promoter, most dramatically in P1. SAHA at 0.5 μM increased H3K9/14 acetylation in regions P1, P3, P4, and NUE. (B) In PCCL3 cells, transient expression of BRAF V600E decreased H3K9/14 acetylation at exon 1 and P1 of the *NIS* promoter while transfection with WT-BRAF had no effect. Histone acetylation status was analyzed by ChIP. The levels of H3K9K14 acetylation are expressed as fraction of the input DNA (material before immunoprecipitation). Threshold cycles (*C*t) were determined for ChIP samples and the input DNA, and the relative amount of immunoprecipitated DNA (% ChIP signal per input DNA) was calculated as 100^2Δ*C*t^. Each bar represents the mean value ±s.e.m. of at least three different experiments. The promoter regions are as presented in Table 1. EXON, exon 1; NUE, nuclear upstream enhancer; CON, control; DOX, doxycycline; rNIS, rat NIS. *P<0.05, **P<0.01.

To investigate specifically the acetylation status of histone at *NIS* promoter, we performed ChIP studies using real-time qPCR primers designed to target several regions of the promoter, including exon 1, P1, P2, P3, P4, and P5 as presented in Table 1. As shown in Fig. 3A, DOX-induced expression of stably transfected BRAF V600E significantly suppressed H3K9/14 acetylation of histone at P1, P2, and P3 in PCCL3 cells. The acetylation was slightly suppressed at exon 1, P4, and P5. No change was seen at the distantly located region of nuclear upstream enhancer (NUE). In contrast and as expected, SAHA increased H3K9/14 acetylation of histone uniformly at all these regions of the NIS promoter. Upon transient transfection of PCCL3 cells for 48 h, BRAF V600E significantly suppressed H3K9/14 acetylation of histone at exon 1 and P1, but the empty plasmid vector or WT-BRAF did not have such an effect (Fig. 3B). In these transient transfection experiments, we did not see significant change in H3K9/14 acetylation of histone at P2, P3, P4, P5, and NUE in PCCL3 cells. One possible explanation is the less robust expression of BRAF and p-ERK in transient transfection than in stable transition of the cells (Fig. 2).

### Suppression of H3K18 acetylation of histone at the *NIS* promoter by BRAF V600E

**Figure 4:**
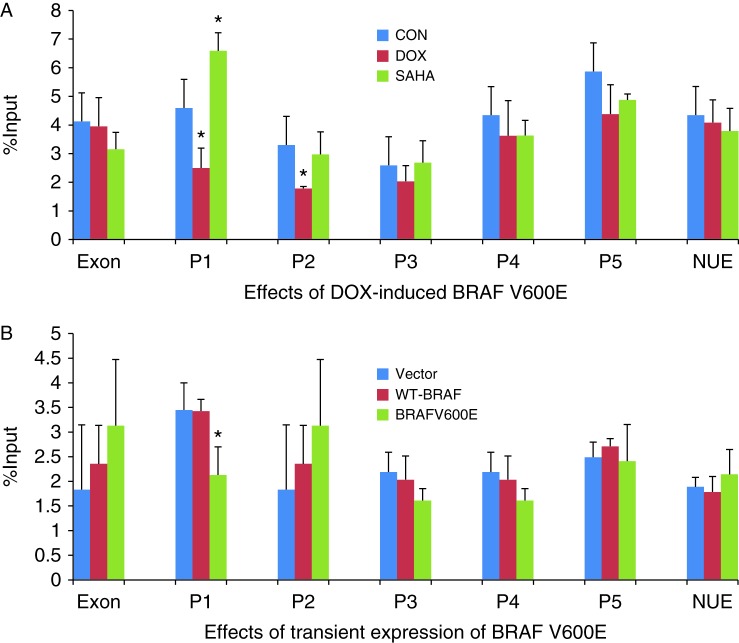
Effect of BRAF V600E on H3K18 acetylation in the rat *NIS* promoter in rat thyroid cells. (A) In PCCL3/BRAF cells, DOX at 1 μg/ml induced BRAF V600E expression after 48 h, accompanied by decrease in H3K18 acetylation in regions P1 and P2 of the *NIS* promoter. SAHA at 0.5 μM increased H3K18 acetylation in regions P1. (B) In PCCL3 cells, transient expression of BRAF V600E decreased H3K18 acetylation at P1 of the *NIS* promoter while transfection with WT-BRAF had no effect. Results of ChIP assays were expressed as the fraction of the input DNA. Each bar represents the mean value ±s.e.m. of at least three different experiments. The promoter regions are as presented in Table 1. Definitions of other symbols are as in Fig. 3. *P<0.05.

As shown in Fig. 4A, DOX-induced expression of stably transfected BRAF V600E significantly suppressed H3K18 histone acetylation at the P1 and P2 regions of the *NIS* promoter in PCCL3/BRAF cells. In contrary, SAHA significantly increased H3K18 histone acetylation at P1. H3K18 histone acetylation was not affected at other regions of *NIS* promoter. Transient expression of BRAF V600E significantly suppressed H3K18 histone acetylation at P1 of the NIS promoter (Fig. 4B). In contrast, transient expression of the WT-BRAF or transfection with the empty vector did not affect H3K18 acetylation at P1. H3K18 histone acetylation at other regions of the *NIS* promoter did not show suppression by BRAF V600E.

### Suppression of total H4 histone acetylation at the *NIS* promoter by BRAF V600E

**Figure 5:**
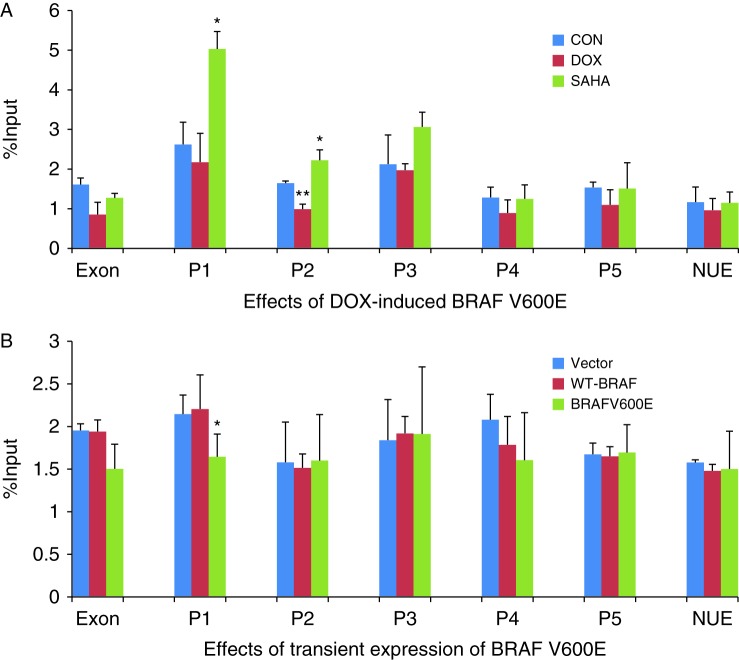
Effect of BRAF V600E on H4 acetylation in the rat *NIS* promoter in rat thyroid cells. (A) In PCCL3/BRAF cells, DOX-induced BRAF V600E expression caused a decrease in H4 acetylation at P2 of the rat NIS promoter and SAHA at 0.5 μM increased H4 acetylation at P1 and P2 of the rat NIS promoter. (B) In PCCL3 cells, transient expression of BRAF V600E decreased H4 acetylation at P1 of the *NIS* promoter while transfection with WT-BRAF had no effect. The levels of H4 acetylation were expressed as fraction of the input DNA. Each bar represents the mean value ±s.e.m. of 3–5 different experiments. The promoter regions are as presented in Table 1. Definitions of other symbols are as in Fig. 3. *P<0.05, **P<0.01.

As shown in Fig. 5A, DOX-induced expression of stably transfected BRAF V600E significantly suppressed total H4 acetylation at the P2 region of the *NIS* promoter in PCCL3/BRAF cells, whereas SAHA significantly increased total H4 acetylation at the P2 region. BRAF V600E also slightly suppressed H4 acetylation at exon 1 and P1, but this was not statistically significant. SAHA also increased total H4 histone acetylation at the P1 region. SAHA slightly increased H4 histone acetylation at the P3 region, but this was not significant. Transient expression of BRAF V600E in PCCL3 cells suppressed total H4 acetylation at P1, whereas transient expression of the WT-BRAF or transfection with the empty vector did not affect H4 acetylation (Fig. 5B). Other regions of the *NIS* promoter were not affected by transient expression of BRAF V600E.

### Suppression of H4K16 histone acetylation of the *NIS* promoter by BRAF V600E

**Figure 6:**
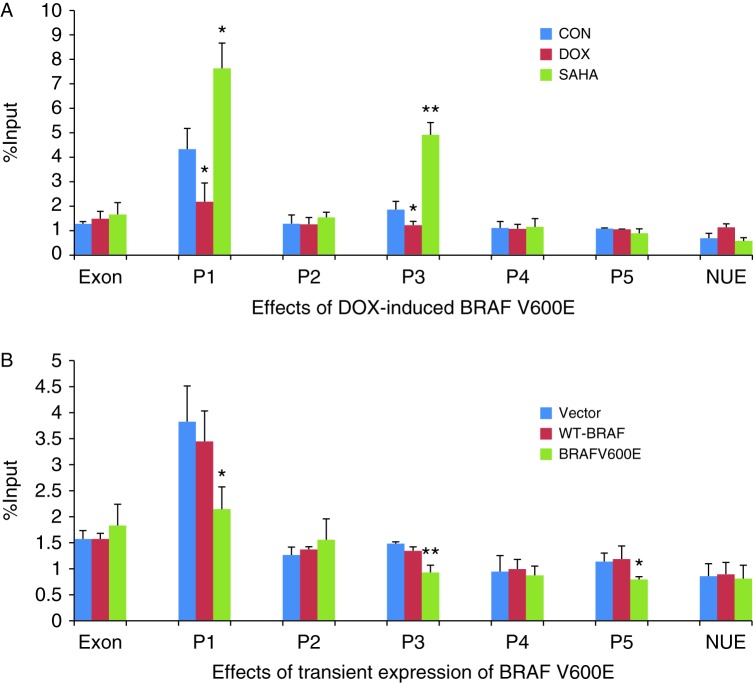
Effect of BRAF V600E on H4K16 acetylation in the rat *NIS* promoter in rat thyroid cells. (A) In PCCL3/BRAF cells, DOX-induced BRAF V600E expression caused a decrease in H4K16 acetylation at P1 and P3 of the rat NIS promoter and increased H4K16 acetylation at NUE of the NIS promoter. SAHA at 0.5 μM increased H4K16 acetylation at P1 and P3 of the rat NIS promoter. (B) In PCCL3 cells, transient expression of BRAF V600E decreased H4K16 acetylation at P1, P3, and P5 of the *NIS* promoter while transfection with WT-BRAF had no effect. The levels of H4K16 acetylation were expressed as fraction of the input DNA. Each bar represents the mean value ±s.e.m. of 3–5 different experiments. The promoter regions are as presented in Table 1. Definitions of other symbols are as in Fig. 3. *P<0.05, **P<0.01.

We also examined specifically the acetylation of K16 of H4 at the *NIS* promoter. As shown in Fig. 6A, DOX-induced expression of stably transfected BRAF V600E significantly suppressed H4K16 acetylation at the P1 and P3 regions of the *NIS* promoter in PCCL3/BRAF cells, whereas SAHA significantly increased H4K16 acetylation at the two regions. Like the increase in the global histone acetylation by BRAF V600E (Fig. 2), DOX-induced expression of BRAF V600E also increased H4K16 acetylation at the NUE region (Fig. 6A). Transient expression of BRAF V600E in PCCL3 cells suppressed H4K16 acetylation at P1, P3, and P5, whereas transient expression of the WT-BRAF or transfection with the empty vector did not affect histone acetylation at these regions of *NIS* promoter in PCCL3 cells (Fig. 6B). Other regions of the *NIS* promoter were not affected by transient expression of BRAF V600E.

### The role of BRAF V600E in modulating histone acetylation of human *NIS* promoter

**Figure 7:**
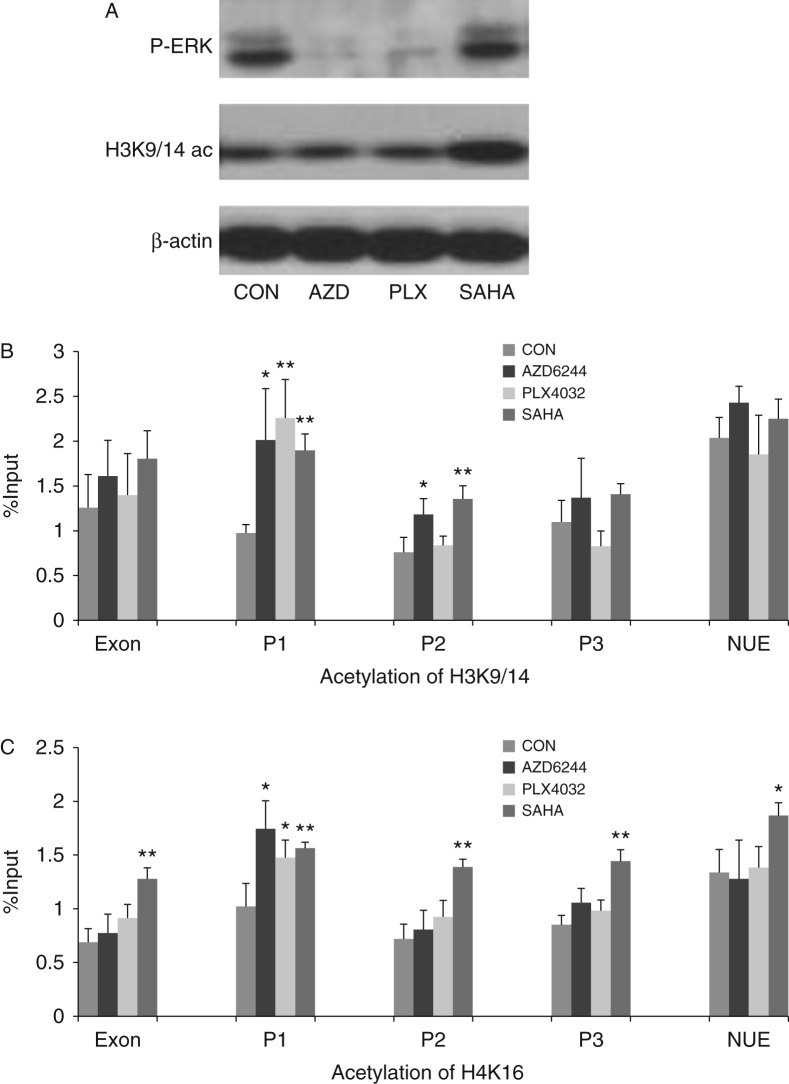
The role of BRAF V600E/MAP kinase pathway in modulating histone acetylation at the human *NIS* promoter in thyroid cancer BCPAP cells. (A) Treatment of BCPAP cells with the MEK inhibitor AZD6244 (AZD) at 1 μM and the BRAF V600E inhibitor PLX4032 (PLX) at 1 μM for 48 h completely suppressed downstream P-ERK and had no significant effect on global H3K9/14 acetylation. The histone deacetylases inhibitor SAHA at 0.5 μM increased global H3K9/14 acetylation in BCPAP cells. (B) Effects of various inhibitors on H3K9/14 acetylation at the human *NIS* promoter. ChIP assay was used to analyze histone acetylation. AZD increased H3K9/14 acetylation at regions P1 and P2, PLX increased H3K9/14 acetylation at region P1, and SAHA increased H3K9/14 acetylation at regions P1 and P2 of the human *NIS* promoter. (C) Effects of various inhibitors on H4K16 acetylation at the human *NIS* promoter. Both AZD and PLX increased H4K16 acetylation at region P1 of the human *NIS* promoter and SAHA increased H4K16 acetylation in all the regions. The levels of histone acetylation were expressed as fraction of the input DNA. Each bar represents the mean value ±s.e.m. of at least three different experiments. The human *NIS* promoter regions are as presented in Table 1. P-ERK, phosphorylated ERK; H3K9/14ac, acetylated H3K9/14; CON, control; AZD, AZD6244; PLX, PLX4032. *P<0.05, **P<0.01. Full colour version of this figure available via http://dx.doi.org/10.1530/ERC-13-0399.

The results presented above obtained from rat thyroid cells indicated an important role of the BRAF V600E/MEK pathway in the modulation of histone acetylation of *NIS* promoter. To confirm that this is the case in human thyroid cancer cells, we used human thyroid cancer cell line BCPAP to test the role of BRAF/MAPK pathway in modulating histone acetylation of human *NIS* promoter. BCPAP cells harbor homozygous *BRAF* V600E mutation (Liu & Xing 2008). As shown in Fig. 7A, the BRAF/MEK pathway was inhibited, as reflected by the suppression of p-ERK, by treatment of BCPAP cells with the MEK inhibitor, AZD6244 (AZD), or the BRAF V600E inhibitor, PLX4032 (PLX). The overall histone H3K9/14 acetylation was not affected by these treatments. SAHA did not affect p-ERK, but it significantly increased the overall acetylation of histone H3K9/14. To test the role of BRAF/MAPK pathway in modulating histone acetylation specifically at the human *NIS* promoter, we similarly treated BCPAP cells with AZD and PLX and selectively examined the acetylation status of two acetylation sites of histone, H3K9/14 and H4K16, at the *NIS* promoter. As for the rat *NIS* promoter, for the human *NIS* promoter we also examined several critical regions, including exon 1, P1, P2, P3, and NUE as presented in Table 1. As shown in Fig. 7B, treatment of cells with AZD resulted in a significant increase in H3K9/14 acetylation at regions P1 and P2. SAHA similarly increased H3K9/14 acetylation at the two regions. Treatment of cells with PLX significantly increased H3K9/14 acetylation at P1 (Fig. 7B). Histone acetylation at other regions of the *NIS* promoter in BCPAP cells was not affected by these treatments. Similarly, AZD and PLX both caused a significant increase in the acetylation of H4K16 at region P1 (Fig. 7C). In contrast, acetylation of H4K16 at other regions was not affected. SAHA increased the acetylation of H4K16 at all the five regions of the human *NIS* promoter. These results suggest that the BRAF V600E/MAPK pathway normally inhibits histone acetylation at the human *NIS* promoter in thyroid cancer cells and removal of this inhibition increased histone acetylation at the *NIS* promoter.

## Discussion

In this present study, we for the first time demonstrated downregulation of histone acetylation specifically at the promoter of both the rat and human *NIS* genes by BRAF V600E. Histone acetylation at the promoter area is a well-established mechanism in the upregulation of genes, which, through chromatin remodeling, opens up the access of gene promoters to transcription factors (Li *et al*. 2007, Dhall & Chatterjee 2011, Horikoshi 2013). Conversely, histone deacetylation causes compacting of chromatin and blocking of gene promoters from binding with transcription factors, resulting in gene silencing. There are multiple lysine residues in the N-terminal tail of histone that are the sites of acetylation, including H3K9, H3K14, H3K18, and H4K16. This study showed that histone deacetylation occurred on all these lysine residues of histones at the *NIS* promoter upon the activation of MAPK by BRAF V600E. Both previous (Puppin *et al*. 2011) and this studies demonstrated an upregulation of global histone acetylation by BRAF V600E, which initially seemed to be puzzlingly against our proposed mechanism in which BRAF V600E-mediated downregulation of *NIS* gene involves deacetylation of histone in thyroid cancer. This puzzle was solved when we looked at the histone acetylation specifically at the promoter of the *NIS* gene and found histone deacetylation at critical regulatory regions of the *NIS* gene. This discordant relations between global histone acetylation and histone deacetylation at a specific gene promoter promoted by an oncogene are similar to another epigenetic phenomenon in human cancer driven by oncogenes, in which global DNA hypomethylation is coupled with hypermethylation of the promoter of tumor suppressor genes (Momparler & Bovenzi 2000), the latter being associated with gene silencing as an important epigenetic mechanism in thyroid tumorigenesis (Xing 2007*b*). Upregulation of *NIS* expression by enhanced histone acetylation has been widely demonstrated in thyroid cells (Puppin *et al*. 2005, Kogai *et al*. 2006, Xu & Hershman 2006, Hou *et al*. 2010, Pugliese *et al*. 2013). Thus, the finding of histone deacetylation of *NIS* promoter by BRAF V600E in this study provides an important epigenetic mechanism for the downregulation of *NIS* gene in BRAF V600E-harboring thyroid cancer.

Histone modifications are conserved in various species including yeast, mouse, rat, and human and their stability is determined mainly by cell types in which the specific regulatory mechanisms control histone modifications (Woo & Li 2012). Therefore, in this study we used rat thyroid cells as a model to study the impact of BRAF V600E/MAPK on histone acetylation of *NIS* and found similar *NIS* promoter deacetylation patterns in rat thyroid cells and human thyroid cancer cells. It has long been known that acetylation of both H3 and H4 tends to occur at promoters areas of individual genes and such histone acetylation is particularly important compared with global histone acetylation in the regulation of individual genes (Vogelauer *et al*. 2000). This is consistent with our present finding on the importance of histone acetylation status of the *NIS* promoter. It is interesting and important to note that among the several regions of *NIS* promoter, P1, P2, and P3 were the most commonly affected with histone deacetylation by BRAF V600E. These regions, particularly PI, of *NIS* promoter are close to the translation initiation site. It is worth noting that histone deacetylation at P1 of the *NIS* promoter occurred virtually with all the histone lysine residues (H3K9, H3K14, H3K18, and H4K16) examined in rat thyroid cells and human thyroid cancer cells. These are among the most important lysine residues for the acetylation of histone affecting gene expression in human cancer (Bjerling *et al*. 2002, Liang *et al*. 2004, Fraga *et al*. 2005). These results suggest that histone acetylation/deacetylation at P1 is particularly important in the regulation of *NIS* expression and thus histone deacetylation at this critical region of *NIS* promoter by BRAF V600E could profoundly affect the expression of *NIS*.

Several important transcriptional factor binding sites have been defined in the *NIS* promoter, including TATA box, AP1, AP2, GR, TTF1, T3Ra, T3R-b, and SP1 binding sites in rat *NIS* promoter (Tong *et al*. 1997) and TATA box, TTF1,AP1, AP2, Sp1, and cAMP response element-binding protein (CREB) binding sites in human *NIS* promoter (Ryu *et al*. 1998). The regions P1 (−297/−107), P2 (−477/−277), and P3 (−678/−452) harbor many of these transcription factor-binding sites and are located in the most active region of the rat *NIS* promoter upstream the translation start site. In rat thyroid cells, DNA fragments (−490 to −118) appears to have the strongest transcriptional activity and a potential TATA box (AATAAAT) is located at −124 to −118 and a TTF1 binding site is located near −480 (Tong *et al*. 1997). Thus, regions P1 and P2 of *NIS* promoter play a critical role in the regulation of *NIS* gene, consistent with the present finding that BRAF V600E/MAPK pathway causes histone deacetylation most profoundly in these regions in rat thyroid cells. In human thyroid cancer cells, treatment with BRAF V600E and MEK inhibitors increased H3K9/14 and H4K16 acetylation mainly at regions P1 (−692/−370) and P2 (−1147/−762) of the human *NIS* promoter, suggesting that BRAF V600E/MEK normally exerts a negative effect on histone acetylation at regions P1 and P2 of the *NIS* promoter in human thyroid cancer cells. A region (−475/−393) within P1 of the human NIS promoter has a strong similarity in DNA sequence (72%) with a region (−196/−114) within P1 of the rat *NIS* promoter (Kogai *et al*. 2006). The DNA sequence of a region (−790/−728) partially involving P2 of the human *NIS* promoter has 63.5% similarity with that of a region (−422/−361) within P2 of the rat *NIS* promoter (Kogai *et al*. 2006). These are consistent with the similar impact of BRAF V600E on the histone acetylation of *NIS* promoter and expression of the *NIS* gene in both rat thyroid cells and human thyroid cancer cells, in this study.

BRAF V600E-promoted loss of expression of *NIS* and other thyroid genes and hence the development of radioiodine refractoriness currently represent a major therapeutic obstacle for thyroid cancer patients. This study not only uncovers an epigenetic mechanism involving histone deacetylation at critical regulatory regions of the *NIS* promoter for BRAF V600E-promoted *NIS* silencing but also provides further evidence supporting our previous proposal to use a combination strategy targeting major signaling pathways and HDAC to restore thyroid gene expression and radioiodine avidity (Hou *et al*. 2010). In this context, the MEK inhibitor AZD, BRAF V600E inhibitor PLX (vemurafenib), and HDAC inhibitor SAHA (vorinostat), which have been approved for the treatment of other human cancers, could reasonably be used as a combination therapy to restore radioiodine avidity for radioiodine treatment of radioiodine-refractory thyroid cancer.

## Author contribution statement

MX conceived and designed the experiments. ZZ, DL & AKM performed the experiments. ZZ & MX analyzed the data. MX contributed reagents/materials/analysis tools. ZZ, ZL & MX wrote the paper.

## Figures and Tables

**Table 1:** qPCR primers of *NIS* promoters for ChIP studies

**Type of NIS**	**Promoter regions**	**Primer sequence** (5′–3′)	**Amplicon size** (bp) **and nucleotide number**
Rat NIS	Exon 1	F: TCACCGAGTCACCTGTCTCCA	124 (−20/104)
		R: TGCACAGCCGACATGAAACT	
	P1	F: CACAACCCTATACGGAACAAG	190 (−297/−107)
		R: TTCGCGCTGCGGATTTATTG	
	P2	F: CAAGCTGCGGAGAAAGGTAGA	200 (−477/−277)
		R: CACAACCCTATACGGAACAAG	
	P3	F: GACAGCCAGGTCAGGACAACATG	226 (−678/−452)
		R: GAGCATCTACCTTTCTCCGCAGC	
	P4	F: ATGCAACACCAGCTCCAG	174 (−1124/−950)
		R: GTGGGCTCATGATGTATATG	
	P5	F: ACCAAGCTTTAGGTGAGGACT	161 (−1874/−1713)
		R: TCTGTGGCCAGTAGAGACTTGA	
	NUE	F: GAATCAGGAGGTTCTACAGT	285 (−2627/−2342)
		R: CTTGATCTTGGAGTCCTGT	
Human NIS	Exon 1	F: CTGGGACTACGGGGTCTTTGC	237 (42/399)
		R: TGCCAGTGGGGCAGGTCCTA	
	P1	F: GAGTGCTGAAGCAGGCTGTGC	323 (−692/−370)
		R: GGGAGCAGCTCGTGATTGTGG	
	P2	F: CTGGCACAGGGCCAACTCTCA	385 (−1147/−762)
		R: TCAGGGTTTCAGGGGACCCATA	
	P3	F: CTGACGCTGTTTCTTTCACCC	296 (−1511/−1216)
		R: GACCACCAGGGAGGTAGAGTC	
	NUE	F: GAGCCCTCAGGCAGTTGCT	238 (−9525/−9287)
		R: ACTCACGTGGAACTGCTTGA	

NIS, sodium iodide symporter; F, forward; R, reverse. Nucleotide number is defined by having the first nucleotide of the translation initiation codon as +1 (Puppin *et al*. 2012, Woo & Li 2012.
